# Peritonectomy of the colonic mesentery. Case report of a new surgical technology

**DOI:** 10.1016/j.ijscr.2021.106369

**Published:** 2021-09-06

**Authors:** Paul H. Sugarbaker

**Affiliations:** Program in Peritoneal Surface Malignancy, Washington Cancer Institute, Washington, DC, USA

**Keywords:** Appendiceal mucinous neoplasms, HIPEC, EPIC, NIPEC, Complete cytoreduction, Mesenteric peritonectomy

## Abstract

**Introduction and importance:**

Malignant peritoneal mesothelioma is a local-regional disease process that requires a comprehensive treatment plan including complete cytoreductive surgery and regional chemotherapy.

**Case presentation:**

Treatments used in our patient began with a complete cytoreductive surgery. This required visceral resections, parietal peritonectomy, peritonectomy of the small bowel and its mesentery, and a peritoneal resection of the colonic mesentery with sparing of the major vasculature of the large bowel.

**Clinical discussion:**

Peritoneal resection of the colonic mesentery and other treatments were performed in the absence of major complications. A 20-day hospitalization was required. The patient shows no internal hernias and no evidence of disease by CT follow-up at 4 years postoperatively. Her quality of life is excellent.

**Conclusions:**

Malignant peritoneal mesothelioma was in the past a disease of limited survival without effective treatment options. Peritoneal resection of the colonic mesentery may be required for complete cytoreduction. A sequence of cytoreductive surgical procedures and regional chemotherapy treatments has made long-term survival possible.

## Highlights

• Malignant peritoneal mesothelioma has treatment options that combined surgery and regional chemotherapy.
• A major surgical challenge is complete mesenteric peritonectomy.
• Complete cytoreduction of the large bowel mesentery requires its resection with sparing major vasculature.
• Long-term survival is possible with low perioperative morbidity and mortality.

## 1 Introduction

The rationale and results of treatment of 802 patients with an epithelial appendiceal neoplasm was recorded by Sugarbaker in 2009 [1]. This is a mucinous neoplasm that disseminates itself widely throughout the abdominal and pelvic space but rarely metastasizes to systemic sites. By analogy, malignant peritoneal mesothelioma has been treated in a similar manner. Epithelial malignant peritoneal mesothelioma throughout its natural history rarely, if ever, develops lymphatic or hematogenous metastases. A treatment plan to exert a maximal local-regional control of this disease has been published [2]. Although the mucinous appendiceal neoplasms may be of large extent throughout the abdomen and pelvis, a relative absence of disease on the small and large bowel and its mesentery is usually observed while the cytoreductive surgery is proceeding. The “motion hypothesis” has been used to explain this absence of disease from the visceral peritoneal surfaces [3]. Malignant peritoneal mesothelioma does not show the sparing of small and large bowel mesentery as is observed with the epithelial appendiceal neoplasms. However, although the small and large bowel mesentery may be completely layered by mesothelioma, the large and small bowel surface itself is spared. The pathophysiology of this frequently observed phenomenon has not been explained to this point in time. However, with this observation as a rationale for treatment, Deraco and colleagues published their experience with a resection of the peritoneal surfaces of the small bowel mesentery. They called this mesenteric peritonectomy [4]. It has been used successfully in carefully selected patients. However, to this point in time involvement of the mesentery of the colon and upper rectum has been managed by total colectomy with ileorectal anastomosis or an end-ileostomy.

In this manuscript, I describe a technique for total removal of peritoneal mesothelioma from the mesentery of the large bowel. This peritonectomy involves a resection of mesentery and peritoneum surrounding the vasculature of the colon and rectum with a sparing of the vessels. A patient who received this total colonic mesenterectomy is presented.

## 2 Materials and methods

Data on this patient was prospectively recorded and then retrospectively reviewed at an academic institution. This research work has been reported in line with the SCARE 2020 criteria [5]. This study was registered as a case report on the www.researchregistry.com website with UIN 6986.

### 2.1 Patient presentation

Fall of 2014: This 51-year-old woman noted a lack of energy and increasing fatigue. Abdominal distention was first noted in mid-2015. Upper endoscopy was performed which showed *H. pylori* which was treated with antibiotics. Colonoscopy was normal. Abdominal and pelvic MRI was read as normal and symptoms were attributed to menopause.

November 2016: A persistent thrombocytosis was noted. An abdominal ultrasound January of 2017 was abnormal.

January 29, 2017: CT of abdomen and pelvis showed ascites. Fluid and enhancing nodules were depicted in the right upper quadrant. The greater omentum had a nodular infiltrate. Small bowel regions were interpreted to be class 1 [6]. Fluid was seen in right and left pararectal fossa. A nodule was present within a minute umbilical hernia. Chest CT was normal.

February 20, 2017: An ultrasound-guided biopsy revealed diffuse malignant epithelial mesothelioma. CA125 tumor marker was normal.

Family history and review of systems was non-contributory.

March 21, 2017: Physical examination was performed. Chest and abdominal exams were normal except for a 1.5 cm nodule within the umbilicus. Pelvic examination was unremarkable.

The treatment plan that was presented to the patient involved cytoreductive surgery, hyperthermic intraperitoneal chemotherapy (HIPEC), early postoperative intraperitoneal chemotherapy (EPIC), placement of an intraperitoneal port and six cycles of combined normothermic intraperitoneal and systemic chemotherapy (NIPEC) [7]. Consent was obtained.

March 31, 2017: A nine-hour cytoreductive surgical procedure with HIPEC was performed. A midline abdominal incision was made with skin tabs so that the umbilicus could be reconstructed [8]. There was a mesothelioma nodule within the umbilicus requiring the umbilicus to be resected. Skin traction sutures were used [8]. The preperitoneal fat above the bladder, the umbilicus, a small midline hernia in the mid-epigastrium, the falciform ligament and the epigastric fat pad were all removed. They were submitted to the pathologist.

**Fig. 1:**
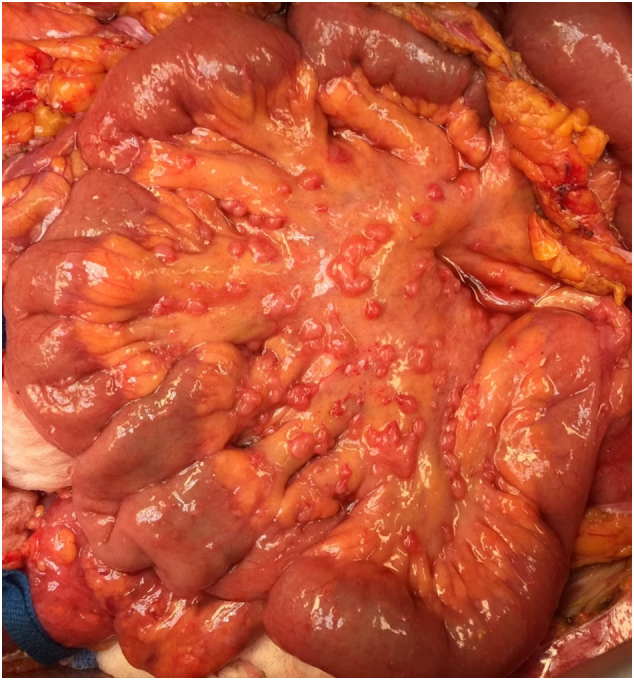
The small bowel mesentery contains malignant peritoneal mesothelioma nodules too numerous to count. The nodules are located on the mesentery of the small bowel. The surface of the bowel itself is relatively spared of the cancer nodules.

The Thompson retractor was inserted. We could explore the abdomen. There was tumor layered out approximately 2 cm thick beneath the right hemidiaphragm. The left hemidiaphragm was clear. The right retrohepatic space was also layered by mesothelioma. The gallbladder was involved, but the stomach was not. The greater and lesser omentum were infiltrated by tumor. The tumor was densely adherent to the transverse colon, especially at the hepatic and splenic flexure. Both pericolic sulci were layered by tumor. The small bowel contained approximately 1000 tumor nodules on its mesenteric surface (Fig. 1). The uterus and ovaries were covered by numerous mesothelioma implants as was the mesentery of the transverse colon and sigmoid colon. It would be necessary to excise these mesenteries and that would include the rectosigmoid colon itself. There was extensive disease in and around the appendix and it would need to be removed. It looked like a complete cytoreduction would be possible, but it would be a long and extensive cytoreductive surgery. Procedures performed included resection of the umbilicus, greater omentectomy, cholecystectomy, lesser omentectomy, peritonectomy of the right upper quadrant, peritonectomy of the small and large bowel mesentery, pelvic peritonectomy, hysterectomy and bilateral salpingo-oophorectomy, left colectomy with low anastomosis, HIPEC, Tenckhoff catheter insertion for EPIC and insertion of an intraperitoneal port for combined intraperitoneal and systemic chemotherapy long-term.

**Fig. 2:**
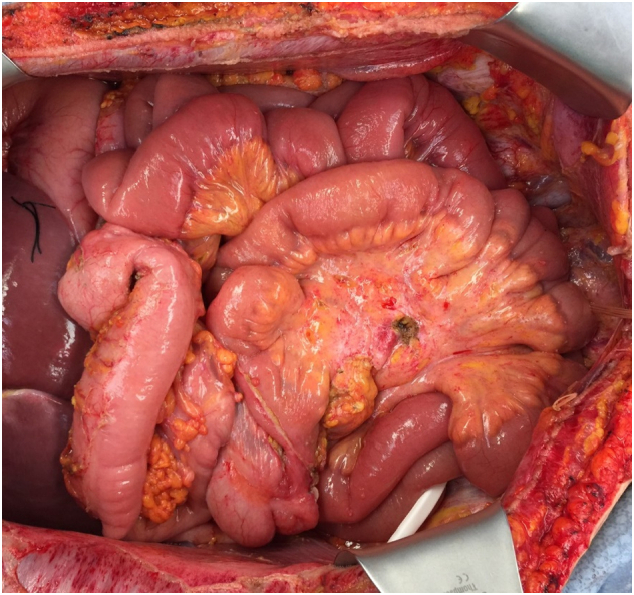
Dissection using the Mayo scissor and limited amounts of electrosurgery are used to clear the small bowel of the extensive tumor nodularity.

**Fig. 3:**
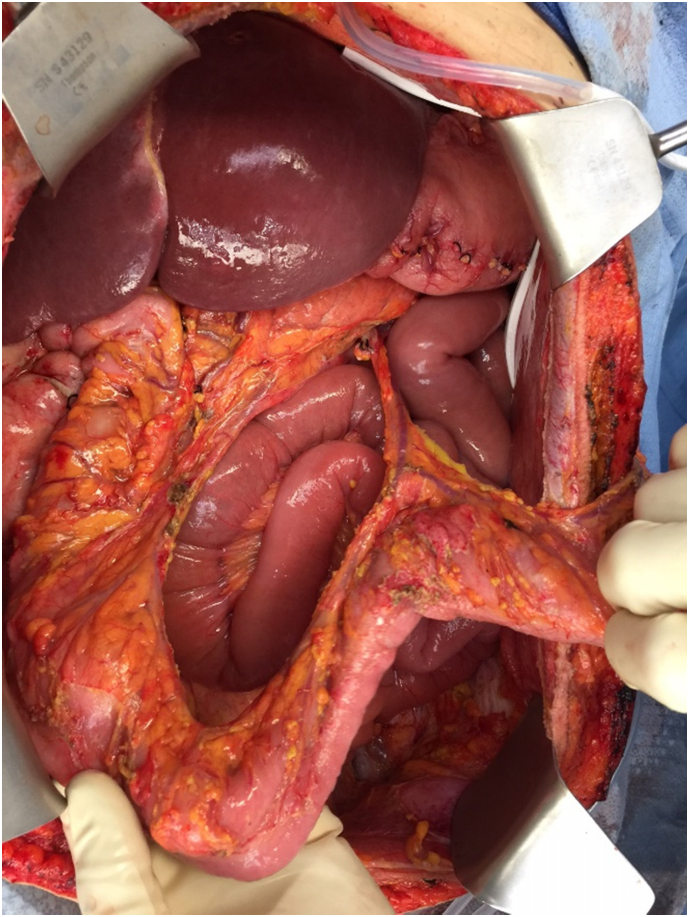
The large bowel mesentery is cleared by resection of the mesentery and sparing of the vasculature of the right colon, transverse colon and descending colon. The rectosigmoid colon and upper rectum are removed as part of the complete pelvic peritonectomy.

The mesothelioma covering the small and large bowel mesenteries presented a special problem [9]. The innumerable nodules on the small bowel mesentery were resected using the curved Mayo scissor (Fig. 2). The mesentery of the colon was resected completely sparing the ileocolic and middle colic vessels. The entire upper rectum and rectosigmoid colon were removed en bloc with the pelvic peritoneum and uterus, tubes and ovaries (Fig. 3). The superior rectal artery was ligated but the left colic artery and vein were spared. Care is taken to spare the intermediate blood supply to the splenic flexure and descending colon. After completion of the HIPEC a two-layer circular stapled anastomosis reinforced by a second layer of silk sutures was used [10]. No diverting ileostomy was performed.

#### 2.1.1 Hyperthermic intraperitoneal chemotherapy

The cytoreductive surgery was followed by HIPEC and EPIC. A pair of curled peritoneal dialysis catheters (Covidien, Mansfield, MA) were used to infuse the chemotherapy solution and four outflow catheters to drain the chemotherapy solution to allow recirculation through the heat pump. Two drugs were administered intraperitoneally in 1.5 l/m^2^ of 1.5% dextrose peritoneal dialysis solution. The two drugs were doxorubicin at 15 mg/m^2^ and cisplatin at 50 mg/m^2^ [11]. The chemotherapy solution was administered as rapidly as possible over approximately 5 min with the infused solution maintained between 41.5 and 43.5 °C within the whole abdomen by a heat pump (Belmont Surgical Instruments, Billerica, MA). A standardized open abdomen technique with manual distribution of the chemotherapy solution was used [12]. Skin edges were elevated on a fixed retractor that formed a rectangle around the open abdomen (Thompson Surgical Instruments, Lansing, MI). A plastic sheet to cover the open abdomen was secured to the retractor by the skin traction sutures. A cruciate incision in the plastic sheet allowed the surgeon's double-gloved hand access to all portions of the abdomen to evenly distribute the heat and chemotherapy solution. The HIPEC treatment was for 90 min. At the initiation of the HIPEC, a continuous infusion of ifosfamide at 1300 mg/m^2^ was started and was continued throughout the 90 min of HIPEC treatments. To prevent uroepithelial damage, 256 mg/m^2^ of sodium methanethiolate (Mesna) was infused intravenously as rapidly as possible 15 min prior to the initiation of HIPEC and 4 h and 8 h later [13]. Following completion of the HIPEC, procedures to repair seromuscular tears, bowel anastomoses, and abdominal closure were performed.

#### 2.1.2 Early postoperative intraperitoneal chemotherapy with paclitaxel

The Tenckhoff catheter and closed-suction drains were maintained after the 90 min of intraoperative chemotherapy. EPIC administration was initiated on the first postoperative day. A 1-liter chemotherapy solution containing paclitaxel at 20 mg/m^2^ was administered intraperitoneally. The carrier solution for the paclitaxel was 6% hetastarch solution (B. Brown, Bethlehem, PA) administered through an infusion pump at 1000 ml/h [14]. At 23 h, the drains and Tenckhoff catheter were unclamped and fluid drained as completely as possible from the peritoneal space prior to instillation of another liter of chemotherapy solution. This procedure was repeated for 5 consecutive days for a total dose of 100 mg/m^2^. A 20-day hospitalization with the patient on total parenteral nutrition through a peripheral intravenous central catheter (PICC) was necessary before return of bowel function.

#### 2.1.3 Long-term combined intraperitoneal pemetrexed and intravenous cisplatin

Prior to closure of the abdomen, an intraperitoneal port (Smiths Medical ASD Inc., St. Paul, MN) was implanted [15]. At 4–6 weeks postoperatively, the intraperitoneal port was accessed. Patients were treated with intraperitoneal pemetrexed at 500 mg/m^2^ in 1 l of 1.5% dextrose peritoneal dialysis solution infused at 1000 ml over 1 h. Following intraperitoneal pemetrexed administration, cisplatin at 75 mg/m^2^ was infused intravenously in 250 ml of normal saline over 120 min. These treatments were repeated for a total of 6 cycles with 3 weeks between each treatment adding approximately 6 months of intensive postoperative management [7]. The patient received all 6 cycles of NIPEC.

#### 2.1.4 Follow-up

CT was performed every 6 months with maximal oral and intravenous contrast. At 4 years, the patient remains disease-free, fully ambulatory with normal bladder and bowel function.

## 3 Discussion

### 3.1 Unique features of malignant peritoneal mesothelioma

Peritoneal mesothelioma has two unique features as compared to other cancers that occur within the abdomen and pelvis. First, this is a malignancy that lacks a primary site. All other cancers start at some specific location or within a specific organ. For example gastric cancer, colon and rectal cancer, ovarian cancer and retroperitoneal sarcoma. Peritoneal mesothelioma arises from the peritoneum but is not limited to any specific site within the abdomen and pelvis. It also progresses on both parietal and visceral peritoneal surfaces. A second unique feature is the progression limited to within the peritoneal spaces. Even as the disease progresses to a terminal state, malignant peritoneal mesothelioma remains confined within the abdomen and pelvis. Direct extension through the tendinous mid-portion of the right or left hemidiaphragm to involve the pleura does sometimes occur, but distant metastases through the blood is very unusual.

It is this second unique feature of peritoneal mesothelioma that causes aggressive local-regional treatment strategies to be successful. Remarkable improvement in survival has occurred as cytoreductive surgery combined with HIPEC has become standard of care for selected patients with epithelial peritoneal mesothelioma [2], [7], [16]. The cytoreductive surgery is a combination of peritonectomy procedures and visceral resections. The goal of this surgery is removal of all disease by visual inspection [17]. The HIPEC, EPIC and NIPEC produces maximal effect when the residual disease is of minimal extent [11]. Removal of disease on visceral peritoneal surfaces which includes the large bowel mesentery is mandatory for a favorable long-term outcome.

### 3.2 Differences in structure of parietal and visceral peritonectomies

Multiple studies have documented that the long-term survival of patients with malignant peritoneal mesothelioma is dependent on the resection of all visible disease from the abdomen and pelvis. Since peritoneal metastases is a disease of the peritoneum itself, this structure must be extensively removed in the majority of patients. Parietal peritonectomy is usually performed without difficulty because of the generous tissues supporting this structure. The parietal peritoneal specimen in need of stripping remains intact so that a complete removal with a margin of heat necrosis provided by high-voltage electrosurgery is possible [17], [18].

In contrast, the visceral peritoneum does not have a fibrous tissue support that allows for the stripping of an intact specimen. In selected patients the tumor itself holds the peritoneal layer intact to allow the mesenteric peritonectomy to be performed [4]. However, except in this unusual situation peritonectomy of the small bowel mesentery is best accomplished by curved Mayo scissor resection of individual nodules and electroevaporation of minute tumor excrescences [17], [18]. The major impediment to complete clearing of small bowel mesentery is often cancer nodules at the junction of small bowel with its mesentery [9]. Removal of these nodules will often result in segmental vascular compromise of the small bowel and a requirement for small bowel resection. In the patient presented the small bowel surface contained minimally invasive nodules that were cleared by scissor dissection with an occasional seromuscular repair after HIPEC.

### 3.3 Peritonectomy of the mesocolon

**Fig. 4:**
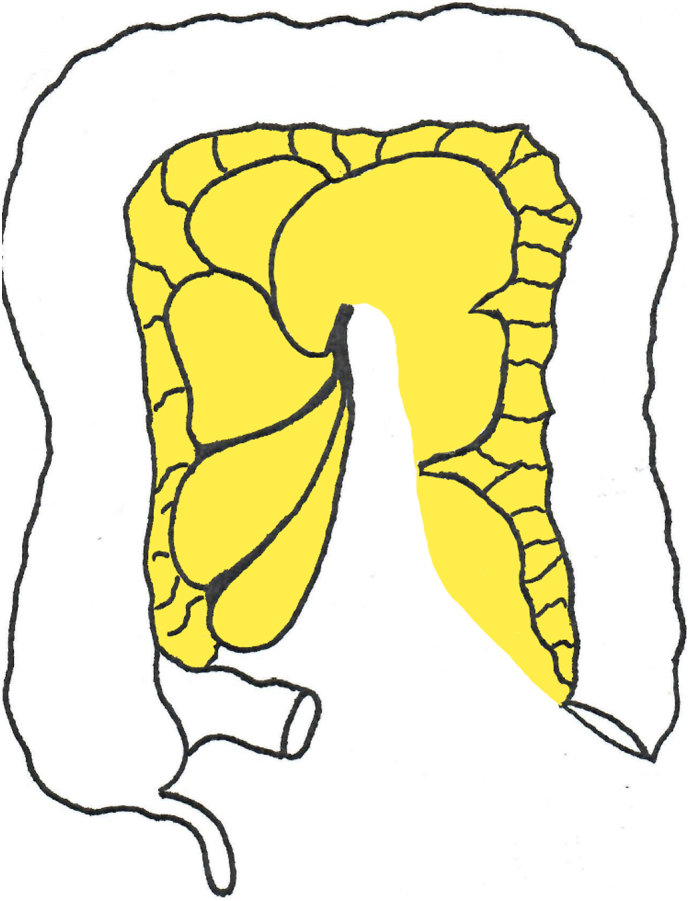
Diagram of the large bowel indicating the avascular spaces between the colonic vasculature which can be removed as a total resection of the colonic mesocolon. Peritoneum on the surface of the marginal vessels is stripped away leaving the vasculature intact.

Peritonectomy of the mesocolon of ascending colon, transverse colon and descending colon presents the surgeon with a dilemma. A formal mesenteric peritonectomy is not possible because there is no mesenteric adipose tissue beneath as in the small bowel mesentery. Scissor dissection will invariably result in multiple perforations through the mesocolon. The technology used successfully on the patient presented is the total resection of the mesocolon with a sparing of its vasculature. The resection of the avascular sections of the mesocolon is illustrated in Fig. 4. This is facilitated by the pelvic peritonectomy, hysterectomy and rectosigmoidectomy which removes the peritoneum of the rectum and rectosigmoid colon. Following HIPEC, the mid-rectal to descending colon two-layer anastomosis is performed.

No closure of the open spaces between colonic vasculature is possible. The mesenteric defect between ileocolic vessels and middle colic vessels is at risk for an internal hernia but the spaces created beyond the middle colic vessels is immediately adjacent to the retroperitoneum with small bowel superficial to its posterior position. No internal hernia has occurred in the patient presented.

## 4 Conclusion

This patient population illustrates that long-term survival is possible in patients with diffuse large volume epithelial type malignant peritoneal mesothelioma. Success begins with cytoreductive surgery that requires visceral resections, mesenteric peritonectomy of the small bowel and its mesentery. Clearing of the mesentery of the colon requires resection of the mesocolon with sparing of the ileocolic and middle colic vessels. HIPEC, EPIC and long-term bidirectional intravenous cisplatin and intraperitoneal pemetrexed follows with access to the peritoneal space by an intraperitoneal port.

## Funding

Data management and secretarial support provided by Foundation for Applied Research in Gastrointestinal Oncology.

## Ethical approval

MedStar Health Institutional Review Board has determined that a case report of less than three [3] patients does not meet the DHHS definition of research (45 CFR 46.102(d)(pre-2018)/45 CFR46.102(l)(1/19/2017)) or the FDA definition of clinical investigation (21 CFR 46.102(c)) and therefore are not subject to IRB review requirements and do not require IRB approval.

## Consent

Written informed consent was obtained from the patient for publication of this case report and accompanying images. A copy of the written consent is available for review by the Editor-in-Chief of this journal on request.

## Registration of research studies

This study was registered as a case report on the www.researchregistry.com website with UIN 6986.

## Guarantor

Paul H. Sugarbaker, MD.

## Provenance and peer review

Not commissioned, externally peer-reviewed.

## CRediT authorship contribution statement

Paul H. Sugarbaker: study concept or design, data collection, data analysis or interpretation, writing the paper.

## Declaration of competing interest

Paul H. Sugarbaker has no conflicts of interest to declare.
